# Grief interventions for people bereaved by suicide: A systematic review

**DOI:** 10.1371/journal.pone.0179496

**Published:** 2017-06-23

**Authors:** Katja Linde, Julia Treml, Jana Steinig, Michaela Nagl, Anette Kersting

**Affiliations:** Department of Psychosomatic Medicine and Psychotherapy, University of Leipzig, Leipzig, Germany; Universita degli Studi di Firenze, ITALY

## Abstract

**Background:**

Adaption to the loss of a loved one due to suicide can be complicated by feelings of guilt, shame, responsibility, rejection, and stigmatization. Therefore people bereaved by suicide have an increased risk for developing complicated grief which is related to negative physical and mental disorders and an increased risk for suicidal behavior. Grief interventions are needed for this vulnerable population. The aim of this systematic review was to provide an overview of the current state of evidence concerning the effectiveness of interventions that focus on grief for people bereaved by suicide.

**Methods:**

We conducted a systematic literature search using PubMed, Web of Science, and PsycINFO for articles published up until November 2016. Relevant papers were identified and methodological quality was assessed by independent raters. A narrative synthesis was conducted.

**Results:**

Seven intervention studies met the inclusion criteria. Two interventions were based on cognitive-behavioral approaches, four consisted of bereavement groups, and one utilized writing therapy. As five of the seven interventions were effective in reducing grief intensity on at least one outcome measure, there is some evidence that they are beneficial. Bereavement groups tend to be effective in lowering the intensity of uncomplicated grief, as are writing interventions in lowering suicide-specific aspects of grief. Cognitive-behavioral programs were helpful for a subpopulation of people who had high levels of suicidal ideation.

**Limitation:**

On average, methodological quality was low so the evidence for benefits is not robust. The stability of treatment effects could not be determined as follow-up assessments are rare. Generalizability is limited due to homogeneous enrollments of mainly female, white, middle-aged individuals.

**Conclusions:**

People bereaved by suicide are especially vulnerable to developing complicated grief. Therefore, grief therapies should be adapted to and evaluated in this population. Prevention of complicated grief may be successful in populations of high risk individuals.

## Introduction

Suicide is a leading cause of death in the U.S. In 2013, 41,149 suicides were reported; that is 12.6 deaths per 100,000 people [1]. Worldwide suicides represent a major public health problem with 804,000 deaths per year—one death every 40 seconds [2].

Losing a loved one is one of the most stressful experiences in life. It has been estimated that for every suicide at least six people experience intense grief [3]. Grief can be seen as a natural response to the loss of a loved one [4]. Within the vast majority of bereaved people, grief intensity decreases within the first year after the loss [5] and a successful adaptation to a life without the deceased is possible without developing any severe physical or mental symptoms [4,6,7]. These people undergo a grief process which can be very painful and exhausting but does not ultimately require treatment [8]. To describe this grief process, the terms “non-pathological grief”, “normal”, and “uncomplicated grief” are used interchangeably in literature. Based on Zisook and Shear’s recommendation [9], we have chosen to use the term “uncomplicated grief” in this systematic review. Basically grief after the loss of a loved one due to suicide resembles grief after a loss by other causes of death. However, people bereaved by suicide differ in terms of suicide-specific aspects of grief that make the bereavement process more complicated [10]. They experience more intense feelings of rejection, a greater need to conceal the cause of the death, and more shame, blaming and social stigmatization than other survivor groups [10–13], even though these reactions are not unique to people bereaved by suicide [14]. A history of mental disorders in the family, prior suicidal attempts of the deceased, and strained family relations [11,12,15,16], as well as less social support after the death [10,12] can also complicate adaption to the loss. Furthermore, people who have found the body of someone who has died by suicide have often described this as being a very traumatic event that evokes flashbacks and intrusive thoughts [17,18] and can further impede the adaption to the loss.

In the following, suicide survivors refer to people who have lost a significant other due to suicide.

The specific circumstances surrounding the loss of a loved one by suicide may contribute to the increased risk of suicide survivors developing a pathological grief reaction [18,19]. Currently, besides “pathological grief”, the terms “persistent”, “traumatic”, “prolonged”, and “complicated” grief are used to describe a condition whereby bereaved people are not able to adapt to or accept the finality of their loss, and the grieving process is complicated, slowed, or halted [4]. For consistency, we have chosen to use the term “complicated grief” in this manuscript. There has been active discussion in recent years about recognizing complicated grief as a distinct mental disorder and establishing diagnostic criteria for it [20–24]. At present, it is already integrated in the Diagnostic and Statistical Manual of Mental Disorders, Fifth Edition [25] as Persistent Complex Bereavement Disorder in section III, a section that contains conditions needing further research. It is also being considered for inclusion in the eleventh revision of the International Statistical Classification of Diseases and Related Health Problems (ICD-11) as Prolonged Grief Disorder [23].

Complicated grief is characterized by intense longing, intrusive preoccupation with the circumstances of the loss, self-blame, avoidance of thoughts or memories of the deceased, avoidance of previously shared activities, and inadequate adaptation to the loss [4]. While complicated grief is estimated to occur in about seven percent of the bereaved in general [26], people bereaved by suicide are at higher risk of developing complicated grief [27–29]. Mitchell et al. [28] reported that, on average, 43% of people bereaved by suicide scored above the caseness threshold of complicated grief one month after the loss. People closely related to the deceased such as their children, spouses and parents experienced nearly twice the level of complicated grief as those who are more distantly related. Three months post-loss De Groot et al. [27] found that 25% of people bereaved by suicide experienced complicated grief compared to 13% bereaved by natural causes of death. Estimates of complicated grief more than one year post-loss vary from 35% in first-degree relatives and spouses [30] to 78% in parents [31]. Complicated grief is associated with several negative health outcomes including cancer, hypertension, cardiac problems, sleep disturbance, reduced quality of life, psychiatric comorbidity including Major Depression and Posttraumatic Stress Disorder, and work and social impairment [24,32–37]. Furthermore, people who suffer from complicated grief are at higher risk for suicidal ideation and behavior [28,38–40] leading to greater mortality rates in this population. Additionally, suicide bereavement itself poses as a risk factor for suicide especially in partners or spouses and parents of people who died by suicide [41,42]. Therefore, suicide survivors are especially in need of interventions aimed at reducing their grief. Studies revealed that suicide survivors report having a great need for professional help in coping with their loss [43,44], particularly in comparison to people bereaved by natural causes of death [27]. In a study by Wilson and Marshall [44], the vast majority of people bereaved by suicide indicated needing professional support in managing their grief. At the same time, only about half of them reported having actually received help from crisis teams, self-help or guided support groups, mental health services, psychiatrists, psychologists, nurses, or other counselors. Although the need for interventions is demonstrably great, little is known about the effectiveness of grief interventions for people bereaved by suicide. Previous reviews have been compromised by various limitations including: an unsystematic review process and a too narrow focus on adults only [12], a too broad focus on studies evaluating effects of interventions on people bereaved by suicide’s general mental health, but not specifically their grief process [45,46]. Furthermore, the last review done before the present one only included studies published before September 2009 [46]. Since then several new studies have been published with results that had yet to be synthesized in a review. A systematic review focusing solely on grief as an outcome variable and not limited to a specific population could contribute to an understanding of effective treatments for people bereaved by suicide.

Schut and Stroebe [47] distinguished three types of bereavement interventions: primary preventive interventions, secondary preventive interventions and tertiary preventive interventions. The first intervention offers professional help to all bereaved persons irrespective of whether intervention is indicated. Secondary preventive interventions are designed for bereaved people at high risk for experiencing a complicated form of grief, i. e. for instance people bereaved due to suicide or homicide. Tertiary preventive interventions are targeted towards bereaved people who are experiencing complications in their grieving process. The purpose of this systematic review is to evaluate the effects of secondary and tertiary interventions on grief for people bereaved by suicide. Hereby, the target outcome grief (in terms of uncomplicated grief, suicide-specific aspects of grief or complicated grief) is taken into consideration.

## Method

### Literature search

The systematic review was conducted in accordance with the PRISMA statement [48]. A systematic literature search for English language papers published from the earliest indexed studies up to November 2016 was conducted using the electronic databases PubMed/Medline, PsycINFO, and Web of Science. The following search terms were used in titles and abstracts: (suicid* OR self-killing) AND (grief OR grieving OR bereave* OR mourning) AND (survivor* OR relative* OR dependant* OR family OR parent OR spouse* OR widow* OR child* OR sibling* OR peer* OR friend*) AND (prevention OR intervention OR postvention OR treatment OR program* OR therapy OR counsel* OR support).Additionally, the reference lists of all relevant papers as well as reviews concerning interventions for the bereaved were scanned.

### Inclusion criteria

To be included in the present systematic review, studies had to meet the following inclusion criteria: (1) publication in a peer reviewed journal, (2) empirical study, (3) inclusion of participants bereaved through suicide, (4) evaluation of any kind of intervention (5) quantitative measure of (complicated) grief, (6) assessments that include at least pre- and post- or follow-up-measurements. Due to the broad scope of the systematic review no restrictions concerning the age of participants or their relationship to the deceased were applied. There were also no limitations on the types of interventions considered.

Articles were excluded if they (1) were not written in English (2) were reviews, case studies, case series, descriptive or qualitative studies, or (3) included suicide survivors as a non-definable subgroup of otherwise bereaved individuals.

After removing duplicates, the first three authors independently screened the title and abstracts for eligible studies. Papers that did not meet the inclusion criteria were excluded. The full text of potentially relevant papers was independently examined by the first three authors. Disagreements were resolved by discussion. If necessary, all authors of this systematic review were consulted until consensus was reached.

### Data extraction

Data extraction from each study meeting the inclusion criteria was done by the first author and independently checked for accuracy by the third author. Disagreements were resolved through discussion. Data was extracted into a data extraction sheet. Variables extracted included: the author(s) of the study, study title and publication year, study design, inclusion and exclusion criteria, number and characteristics of participants (gender, age), characteristics of bereavement outcomes (time since bereavement, relationship to the deceased), characteristics of the intervention and of comparison groups (individual or group intervention, duration, frequency of contact, kind of control group), time points of assessment, outcome measures, drop-out rates, statistical analyses applied, main results, and information necessary for evaluating methodological quality (e.g. confounders).

### Quality assessment

Methodological quality was assessed independently by the first and fourth author using the Quality Assessment Tool for Quantitative Studies [49]. The studies were rated in relation to the following six components: selection bias, study design, confounders, blinding, data collection method, withdrawal and dropouts. Values between 1 “strong”, 2 “moderate”, and 3 “weak” were assigned. Disagreements were discussed with all authors until consensus was reached. The results of the quality assessment were used to describe the overall quality of the included studies and to score the quality of each individual study.

### Data synthesis

The included studies were highly diverse in terms of study design, characteristics and intensity of interventions, as well as outcome measures. Therefore, based on recent guidance [50], a narrative synthesis of the data was conducted instead of pooling data for a meta-analytic approach. Similarities and differences between study findings were analyzed with regard to study characteristics, recruitment criteria, characteristics of participants and bereavement, characteristics of the intervention, outcome measures, and methodological quality. Studies were grouped according to whether they had an inactive or active comparison group, and whether they had a focus on uncomplicated grief, suicide-specific aspects of grief, or complicated grief.

## Results

### Study characteristics

**Fig 1:**
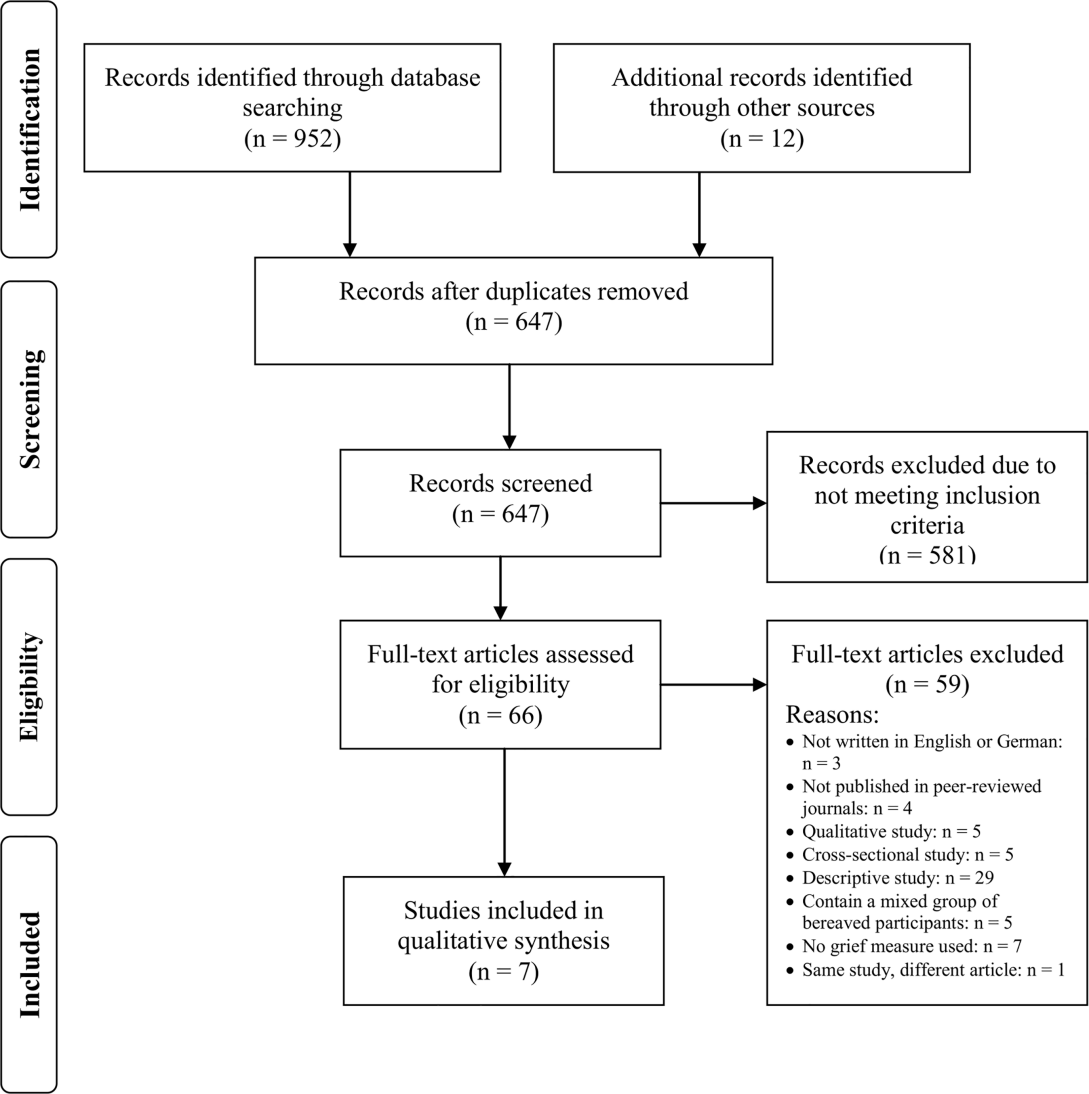
PRISMA flow diagram.

In total, *N* = 952 titles and abstracts were identified using electronic databases. Of those, 305 were excluded because they were duplicates, and 647 were screened by the first three authors for inclusion in the systematic review. Of these, 580 publications were excluded due to not meeting the inclusion criteria. An additional 12 publications were identified through screening reference lists of relevant papers and reviews. In total, 67 publications were screened full-text by the first three authors and seven studies met the eligibility criteria (Fig 1). One of those studies was described in two articles [30,51] and only the most recent article which incorporates the older one was included in this systematic review.

The included studies were published between 1992 and 2014. Four studies were conducted in the USA [52–55], one in the Netherlands [51], one in Canada [56], and one in Belgium [57].

### Recruitment criteria

In four of the included studies, participants were recruited both through self- and professional referral [51–54], two used self-referral only [56,57], and one used researcher referral [55].

Overall, the studies had few inclusion and exclusion criteria (Table 1). Of those who reported inclusion criteria (5/7), two required the loss of a loved one, one the loss of a first-degree relative or spouse, and one the loss of a spouse to suicide. One study was explicitly restricted to participants older than 15 years [51], and three studies were restricted to participants older than 18 years [53,56,57]. Three studies were restricted to a specific time since bereavement varying between a loss within the past eight weeks [51] and two years [55,57]. None of the studies restricted study participation according to the intensity of grief, presence of complicated grief, or other mental health problems. Only Kovac and Range [55] restricted study participation to those survivors who stated that they had been very close to the deceased and very upset by their death.

**Table 1:** Characteristics of included studies.

	Barlow et al. (2010)	Constantino & Bricker (1996)	Constantino et al. (2001)	De Groot et al. (2010)	Farberow (1992)	Kovac & Range (2000)	Wittouck et. al (2014)
Inclusion criteria	1. Clients ≥18 years 2. Clients of CMHA^a^ Suicide Service; Peer supporters: Time since bereavement ≥ 2 years	NR	1. Survive the suicide of a spouse 2. Clients ≥18 years English speaking	1. First degree relatives or spouses of people who had died by suicide Loss occurs within the past 8 weeks	NR	1. Loss of a loved one to suicide in the past two years 2. Close to the deceased Upset by the death	1. Loss of a loved one to suicide in the past 3 months to 2 years 2. Clients ≥18 years, Dutch speaking
Sample size	Total: 19	Total: 32	Total: 60	Total: 122	Total: 82	Total: 42	Total: 83
	I: 19, C: None	I:16, C:16	I: NR, C: NR	I: 68, C: 54	I: 60, C: 22	I: 20, C: 22	I: 47, C: 36
Age (years)	M = 46.9 (SD = 10) Range: 26 to 66	M = 43	Range: 24–70	NSI: M = 43 (SD = 14.1), SI: M = 42 (SD = 12.1)^b^	77% between 20 and 49 Range: 10 to over 60	M = 24.0, SD = 7.3; Range: 18–46	M = 48.6, SD = 13.3
Men N (%)	3 (18.8)^c^	Minority^d^	10 (21.3)^e^	40 (32.8)	23 (28.0)	9 (21.4)	20 (24.1)
Women N (%)	13 (81.3)^c^	Majority^d^	37 (78.7)^e^	82 (67.2)	59 (72.0)	33 (78.6)	63 (75.9)
Relationship to deceased	Mixed: wife, husband, father, son, sister, common-law-husband^d^	Spouses or partners^f^	Spouses or partners^f^	Mixed: 29.5% spouse, 23.8% parent, 22.1% child, 17.2% sibling, 7.4% in-law/other	Mixed: spouse, sweetheart, parent, child, sibling,other ^e^	Not reported	Mixed: 24.1% partner, 9.6% parent, 39.7% child, 18.1% sibling, 8.5% other
Time since bereavement	6 weeks to 20 years, 75% reporting deaths within the past 5 years	Not reported.	M = 10.9 (SD = 8.7), range: 1 to 27 month	Not explicitly stated; assumed to be less than 2.5 months	Less than 3 to over 24 months, 77% reporting deaths within the past 8 months	I: 13.3 (SD = 9.32) monthsC: 12.0 (SD = 6.5) months	M = 11.0 (SD = 6.1) months, Range: 3 months to 2 years
Characteristics of intervention	Peer support program	Bereavement group postvention	Bereavement group postvention	Family-based grief counseling program using cognitive- behavioral therapy	Bereavement group program	Writing therapy	CBT-based psychoeducational intervention
Type of intervention	Group intervention	Group intervention	Group intervention	Group intervention	Group intervention	Individual	Individual or group intervention
Duration	4 months	8 weeks	8 weeks	NR	8 weeks	2 weeks	NR
Frequency of contact	Not reported. Average duration 96.8 minutes.	Once a week with a duration of 1.5 hours	Once a week with a duration of 1.5 hours	4 sessions every 2 to 3 weeks with a duration of 2 hours	Once a week with a duration of 1.5 hours	4 sessions with a duration of 15 minutes	4 times with a duration of 2 hours
Implemented by	Trained volunteer peer supporters	Trained leader with a master’s degree in mental health nursing	Trained leader with a master’s degree in mental health nursing	Experienced psychiatric nurse	Mental health professional and trained survivor	Researcher	Clinical Psychologist
Characteristics of Comparison group	No comparison group	Social group postvention	Social group postvention	No intervention	No intervention or those who dropped out after one session	Writing Group	No intervention

I = Intervention group. C = Comparison group. NR = not reported. SI = Suicide Ideators. NSI = Non Suicide Ideators.

^a^ Canadian Mental Health Organization.

^b^ No information about total sample available.

^c^ Refers to *n* = 16 participants who are completers.

^d^ No details reported.

^e^ Refers to *n* = 47 participants who are completers.

^f^ No frequencies reported.

### Characteristics of participants and bereavement

The sample size varied from 19 to 122 participants (Table 1) with the majority of studies (6/7) including fewer than 85 participants. The majority of participants in all of the studies were women. The proportion of men varied from 18.8 to 32.8 percent (Table 1). Except for the study by Kovac and Range [55], which included students with a mean age of 23.98 years, in all the other studies, participants were, on average, middle-aged adults (mean age range from 43.00 to 48.6 years). None of the studies focused on children, adolescents, or older adults. Where information about ethnic data was available (3/7), the majority of participants were Caucasian.

Six of seven studies gave information about the relationship to the deceased (Table 1). In four of these studies [51,54,56,57], the participants had diverse relationships (e.g., children, parents, partner) to the deceased, and in two studies [52,53], only spouses or partners were included. Where reported (Table 1), the most frequent relationships were partners, children, and parents. None of the studies focused on mental health care professionals such as psychiatrists or psychotherapists or people with other occupations who are at higher risk of knowing someone who has died by suicide. Average time since bereavement varied between studies from less than 2.5 months [52,53] to five years [56]. The Barlow et al. [56] study was an outlier in this case as time since bereavement in all the other studies was less than or around 12 months.

Four of seven studies provided at least some information about the deceased. In the two studies that reported the sex of the deceased [51,56] the majority were men. Mean age of the deceased was assessed in four studies and varied from 28.90 years (SD = 10.84) for the whole sample in the study by Kovac and Range [55] to 46.00 (SD = 15.2) years for the comparison group in the study by de Groot et al.[51]. Only one study reported the method of suicide [53]. In this study, most frequently used were gunshot and carbon monoxide poisoning.

### Characteristics of interventions

The majority of studies (5/7) evaluated group interventions, one studied a mixture of individual and group interventions [57], and one assessed an individual intervention [55] (Table 1). Where reported, the duration of the interventions ranged between two [55] and 16 weeks [56] with most of the interventions lasting about eight weeks. In the majority of the studies (6/7), single intervention sessions lasted between 90 and 120 minutes, while the sessions in the study by Kovac and Range [55] only lasted 15 minutes. The frequency of contact varied from four times within two weeks [55] to four times every two to three weeks [51]. The intervention sessions usually took place once a week [52–54].

Except for two studies [55,56], all interventions were delivered by mental health professionals or a combination of mental health professionals and a trained survivor (Table 1). Of these, only one was delivered by a clinical psychologist [57]. In the two other studies, the researcher [55] and a trained survivor delivered the intervention [56]. No study explicitly stated having used an intervention manual. Only one study stated that supervision was provided for those who delivered the intervention [51].

One study evaluated the effectiveness of the intervention without a comparison group [56] and three studies compared the intervention to an inactive comparison group [51,54,57]. Two interventions were based on cognitive behavioral therapy [51,57], one was a peer support program [56], and one a bereavement group program [54]. Three studies compared the effectiveness of an intervention to an active comparison group [52,53,55]. One compared an emotional writing condition to a neutral writing condition [55], and two compared a bereavement group postvention to a social group postvention [52,53].

Five of seven studies explicitly mentioned the theoretical background of their intervention. In two studies, the intervention emphasized the 12 therapeutic factors of group therapy formulated by Yalom, 1995 [52,53]. In two other studies, the intervention was based on the conceptualization of complicated grief by Boelen et al., 2006 [51,57], and one intervention was based on the writing paradigm formulated by Pennebaker, 1986 [55].

### Outcomes measures

Various measures were used ranging from a self-generated single item evaluating grief intensity [54] to seven different standardized grief measures. The standardized grief measures and their psychometric properties are described in Table 2. The majority of instruments were self-report questionnaires but one was a structured interview for clinical use. Four instruments had a focus on uncomplicated grief, one on suicide-specific aspects of grief, and only two on complicated grief. Of those, the Inventory of Traumatic Grief (ITG) was used in a scale format to assess the intensity of complicated grief. The Traumatic Grief Evaluation of Response to Loss (TRGR2L) was used to diagnose complicated grief in participants based on consensus criteria of complicated grief [51]. Only two studies included not only post intervention but also follow-up assessments at intervals ranging from six [55] to 12 months [53].

**Table 2:** Description of grief measures.

Instrument (author, year of publication)	Included in	Type/ number of items/ scale/ reference period	Scales /Subscales	Reliability/Validity	Focus
Grief Cognitions Questionnaire (GCQ) (Boelen et al., 2003)	Wittouck et al. (2014)	questionnaire/38/ 6-point rating scale/NR	9 subscales: global negative beliefs about the self, the world, life, future, negative cognitions about self-blame, other people`s response s after the loss, appropriateness of grief reactions, cognitions reflecting the importance of cherishing the pain of the loss, threatening interpretations of one`s own reactions to the loss	.81 ≤ α ≤ .95/ Construct, convergent, discriminative validity shown [58]	Uncomplicated grief
Grief Experiences Inventory (GEI) (Sanders et al., 1985)	Constantino & Bricker (1996); Constantino et al. (2001)	questionnaire /135/ 2-point rating scale/ NR	1 total scale and 9 subscales: Despair, Anger/Hostility, Guilt, Social Isolation, Loss of Control, Rumination, Depersonalization, Somatization, Death Anxiety	.52 ≤ rtt ≤ .85/ NR [59]	Uncomplicated grief
Grief Experience Questionnaire (GEQ) (Barrett & Scott, 1989)	Kovac & Range (2000)	questionnaire/55/ 5-point Likert scale/the first two years after the death	1 total scale and 11 subscales: Somatic reaction, general grief reaction, search for explanations, loss of social support, stigmatization, guilt, responsibility, shame, rejection, self-destructive behavior, unique reactions	.76 ≤ α ≤ .97/ Discriminative validity shown [60]	Suicide-specific aspects grief
Grief Recovery Questionnaire (GRQ), (Lehmann et al. 1986; Lehmann et al., 1987)	Kovac & Range (2000)	questionnaire^a^ /8/ 5 items 9-point Likert scale, 3 items dichotomous format/ NR	1 total scale	Not reported originally but Kovac & Range (2000) reported *α* = .83/ NR	Uncomplicated grief
Hogan Grief Reaction Checklist (Hogan et al., 2001)	Barlow et al. (2010)	questionnaire/61/ 5-point Likert scale/past two weeks, including today	6 subscales: Despair, Panic Behavior, Personal Growth, Blame and Anger, Detachment, Disorganization	.79 ≤ α ≤ .90; .56 ≤ rtt ≤ .85/ Construct, convergent & divergent validity shown [61]	Uncomplicated grief
Inventory of Traumatic Grief (ITG), (Prigerson et al., 1995; Dutch Version Boelen et al. 2003)	De Groot et al., (2010); Wittouck et al. (2014)	questionnaire/29/ 5-point Likert scale/ the last months	1 total scale: maladaptive grief	α = .94, rtt = .92/ Construct, discriminative, concurrent validity shown [62]	Complicated grief
Traumatic Grief Evaluation of Response to Loss (TRGR2L) (Prigerson & Jacobs, 2001)	De Groot et al., (2010)	structured interview/NR/ 5-point rating scale for frequency and intensity of each symptom/ NR	Diagnosis of maladaptive grief reaction based on consensus criteria of traumatic grief. If at least one item was scored above two on both the frequency and intensity rating a maladaptive grief reaction was present.	Kappa = 0.71/ Criterion-related validity shown	Complicated grief

NR = Not reported, α = Cronbachs`Alpha, rtt = test-retest reliability.

^a^ developed as an interview.

### Methodological quality

Table 3 provides an overview of the assessment of methodological quality for each study. Studies were rated in relation to selection bias, study design (including randomization), confounders, blinding, data collection method, withdrawal, and dropouts. Overall, study quality was weak. No study received a strong rating on any of the six components of methodological quality. The biggest problems were selection bias and blinding as none of the studies recruited a representative sample, and none described the outcome assessor or the participants as having been blinded. Additionally, two studies used a non-randomized study design [54,56] and only one study [51] based their analysis on intention-to-treat analyses.

**Table 3:** Methodological quality of included studies.

	Barlow et al.(2010)	Constantino & Bricker (1996)	Constantino et al. (2001)	De Groot et al. (2010)	Farberow (1992)	Kovac & Range (2000)	Wittouck et. al (2014)
**Selection Bias**	**Weak**	**Weak**	**Weak**	**Weak**	**Weak**	**Moderate**	**Weak**
Representative?	Not likely	Not likely	Not likely	Somewhat likely	Not likely	Somewhat likely	Not likely
Percentage agreement^1^	60–79%	80–100%	Can`t tell	Less than 60%	Can`t tell	60–79%	80–100%
**Study Design**	**Moderate**	**Strong**	**Strong**	**Weak**	**Moderate**	**Strong**	**Strong**
Study Design	Cohort (one group pre and post)	RCT	RCT	Secondary analyses of an RCT	Cohort analytic (two groups pre and post)	Controlled Clinical trial	RCT
Described as randomized?	No	Yes	Yes	Yes	No	Yes	Yes
Method of randomization described?	N.A.	Yes	Yes	Yes^2^	N.A.	No	Yes
Method appropriate?	N.A.	Yes	Yes	Yes	N.A.	N.A.	Yes
**Confounders**	N.A.	**Strong**	**Strong**	**Strong**	**Weak**	**Weak**	**Strong**
Important pre-intervention differences?		No	No	Yes	Yes	Yes	Yes
Percentage confounders controlled for?		N.A	N.A	0–100%	Less than 60%	Less than 60%	80–100%
**Blinding**	**Weak**	**Weak**	**Weak**	**Weak**	**Weak**	**Strong**	**Weak**
Outcome assessor described as blinded?	No	No	No	No	No	Yes	No
Participants blinded?	No	Can`t tell	Can`t tell	Can`t tell	Can`t tell	Yes	Can`t tell
**Data Collection Method**	**Strong**	**Weak**	**Weak**	**Strong**	**Weak**	**Weak**	**Strong**
Measures valid?	Yes	Can`t tell	Can`t tell	Yes	No	No	Yes
Measures reliable?	Yes	Yes	Yes	Yes	No	Yes	Yes
**Withdrawals and Dropouts**	**Weak**	**Strong**	**Moderate**	**Strong**	**Weak**	**Moderate**	**Strong**
Number and reasons reported per group?	No	Can`t tell	No	Yes	Can`t tell	Yes	Yes
Percentage completing study?	Less than 60%	80–100%	60–79%	80–100%	Can`t tell	Less than 60%	80–100%
**Number of strong ratings**	**1/6**	**3/6**	**2/6**	**4/6**	**0/6**	**2/6**	**4/6**
**Intention-to-treat analysis**	No	No	Can`t tell	Yes	Yes	No	No

^1^ Refers to the % of subjects in the control and intervention group that agreed to participate in the study before they were assigned to intervention or control group.

^2^ described in De Groot et al. (2007), RCT = randomized controlled trial

### Study findings

#### Studies without a comparison group

**Fig 2:**
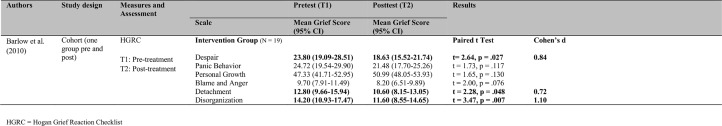
Summary of the included studies without a comparison group.

One study [56] evaluated a four-month suicide bereavement peer support program within a one-group pre-post design (*N* = 19). The majority of participants reported deaths within the past five years. The intervention consisted of personal meetings or telephone conversations between peer supporters and participants. No further information is provided regarding the intervention. From pre- to post-assessment, three out of six subscales measuring uncomplicated grief (Despair, Detachment, and Disorganization) indicated significant reductions (see Fig 2). Due to the uncontrolled study design, it is impossible to attribute these changes solely to the effects of the intervention. The study was also limited by the small sample size and a high study drop-out rate of nearly 50 percent. Overall, methodological quality was low (Table 3).

#### Studies comparing the intervention to an inactive comparison group

**Fig 3:**
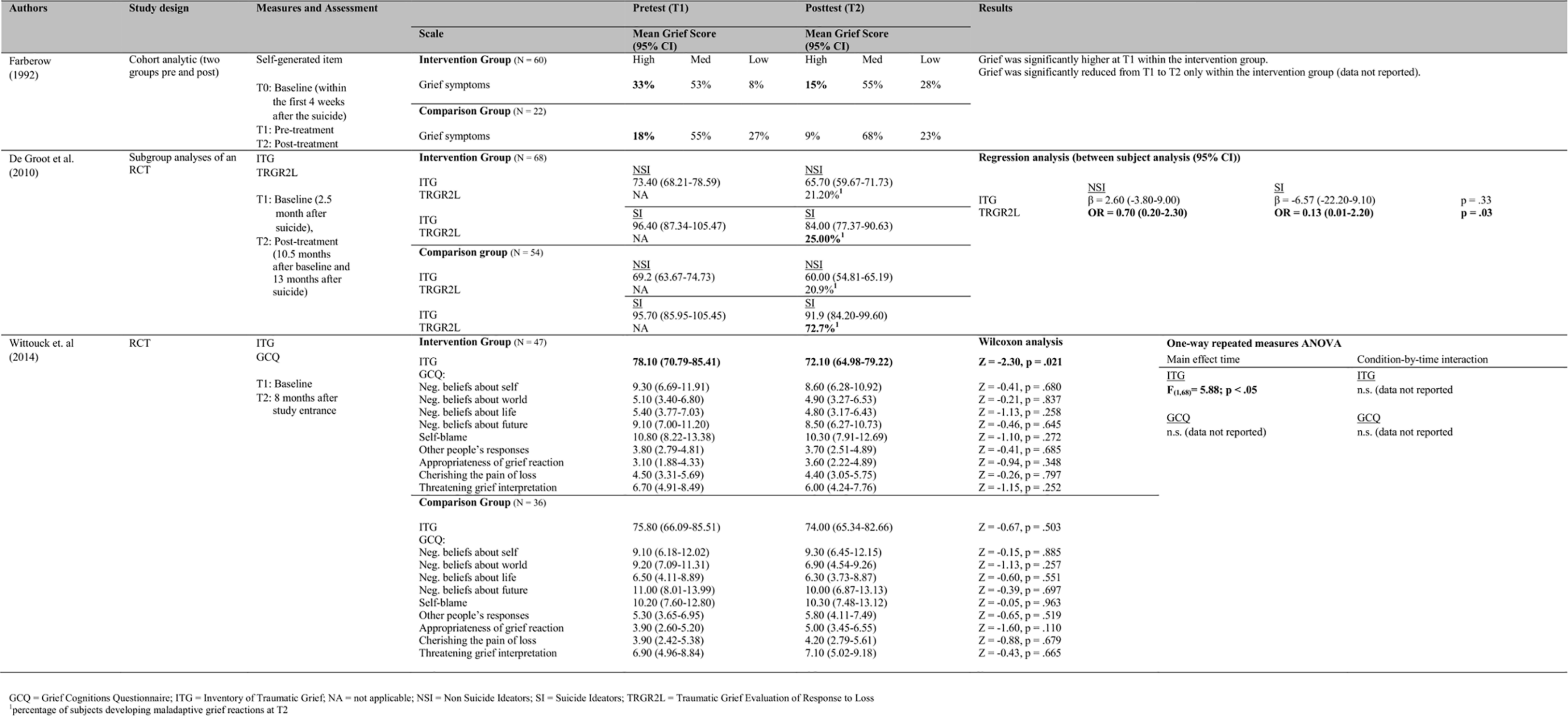
Summary of the included studies with an inactive comparison group.

Three studies compared the intervention to an inactive comparison group. In two studies, the effectiveness of the intervention was evaluated with regard to uncomplicated grief [54,57] and in two studies with regard to complicated grief [51,57] (see Fig 3).

With regard to uncomplicated grief, study findings were mixed (Fig 3). Faberow et al. [54] evaluated an 8-week “Survivors After Suicide” bereavement group program (*N* = 82) and found positive intervention effects. Time since bereavement varied between three and 24 months with 77% of participants reporting deaths within the past eight months. The intervention consisted of a group therapy developed to provide support in dealing with difficult emotions and coping with grief. Uncomplicated grief was significantly reduced from pre- to post-assessment in the intervention but not in the comparison group. Validity is limited due to the non-randomized study design and the lack of controlling for pre-treatment differences in grief intensity between the groups. Furthermore, the results are only reported as changes in percentages but no total scores and method of analyses are described. Therefore results cannot be replicated.

No intervention effect was found in a methodologically sound randomized-controlled trial (RCT) by Wittouck et al. regarding uncomplicated grief [57]. They evaluated an intervention based on cognitive-behavioral therapy (*N* = 83). Time since bereavement was, on average, eleven months. The intervention comprised psychoeducation regarding aspects of suicide (illustrating the suicidal process and explaining a comprehensive explanatory model of suicidal behavior), aspects of bereavement in general and specific to suicide (including myths regarding content, course and cultural context of grief) and coping (discussing the dual-process model of coping with bereavement [63]). No significant effect of the intervention was found using a generalized grief reactions measure (Fig 3).

With regard to the intensity of complicated grief, study findings were ambiguous. Of the two methodologically sound randomized controlled studies [51,57], one did not find any treatment effects for bereaved persons in general [57] and one found a treatment effect but only for a subgroup of bereaved individuals who suffered from suicidal ideation before treatment [51]. The study finding no intervention effect is the RCT by Wittouck et al. [57] which was described above. Besides using a generalized grief reactions measure they also applied a measure for complicated grief symptoms but found again no significant effect of the intervention. In the RCT done by De Groot et al. [51], a family-based grief counselling program based on cognitive-behavioral therapy was evaluated (*N* = 122). Time since bereavement was less than 2.5 months. This time frame was chosen in order to intervene before negative beliefs become fixed. Among other things, the intervention consisted of cognitive restructuring, consolidation of support, family grief and communication, and improving problem solving. In their initial analyses, no significant differences at the post-assessment were found between the intervention and comparison groups with or without controlling for covariates. Later they reanalyzed the data and evaluated the program in subgroups of participants with (22%) and without suicidal ideation (78%). Although the self-report questionnaire indicated no significant intervention effects on complicated grief symptoms in participants with or without suicidal ideation, the intervention was shown to be effective on a diagnostic level of complicated grief as assessed in clinical interviews (Fig 3). The analyses showed that, among participants with suicidal ideation, those who received the intervention were diagnosed with complicated grief at post-assessment significantly less frequently than participants who did not receive the intervention. Within the intervention group, a nearly equal percentage of participants with and without suicidal ideation (20.9 vs. 21.2%) developed a maladaptive grief reaction at post-assessment, whereas within the comparison group, 72.7 percent of participants with suicidal ideation developed a maladaptive grief reaction compared to 25.0 percent of participants without suicidal ideation.

#### Studies comparing the intervention to an active comparison group

**Fig 4:**
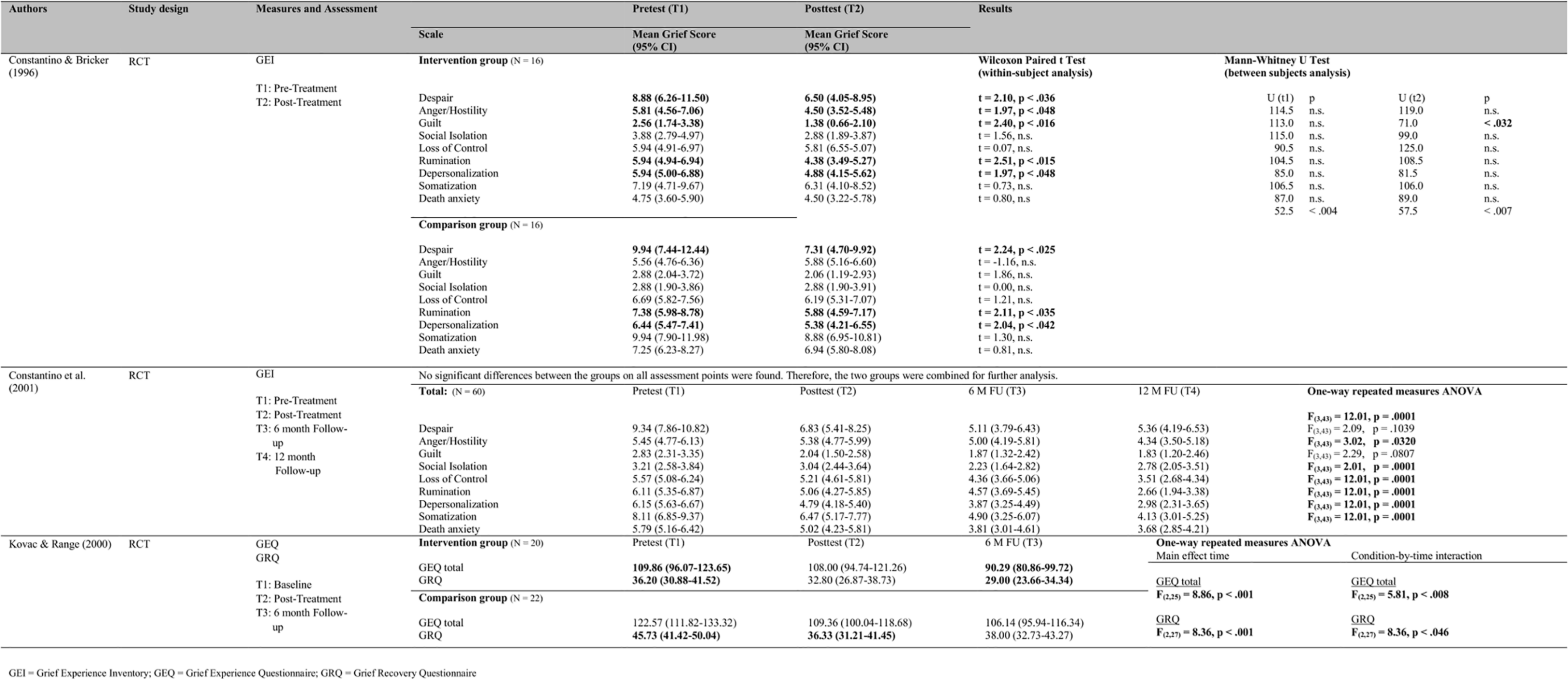
Summary of the included studies with an active comparison group.

Three studies compared an intervention to an active comparison group [52,53,55]. All of the studies evaluated the effectiveness of interventions with regard to uncomplicated grief and one also focused on suicide-specific aspects of grief [55] (Fig 4).

With regard to uncomplicated grief, study findings were mixed. Whereas one study found that the intervention was more effective than the active comparison group [52], two studies found no significant differences between the intervention and active comparison groups [53,55]. In an RCT, Constantino and Bricker [52] compared the effects of an eight-week bereavement group to an eight-week social group postvention (*N* = 32). Time since bereavement was not reported. The bereavement group emphasized the twelve curative factors of group psychotherapy as formulated by Yalom 1985, whereas the social group promoted the principles of socialization, recreation, and leisure. Out of nine different aspects of uncomplicated grief, three aspects (Despair, Rumination, Depersonalization) were significantly reduced in both groups and only one (Anger/Hostility) was significantly reduced from pre- to post-assessment in the intervention group but not in the active comparison group (Fig 4). The later RCT done by Constantino et al. [53] was a replication of the study done by Constantino and Bricker [52]. The same study design, measures, and interventions were used but the sample size was twice as large (*N* = 60). Time since bereavement was, on average, 10.91 months with a range from one to 27 months. Besides pretest and posttest assessments there were also 6- and 12-months follow-up assessments. No significant differences between the intervention and active comparison group were found since both groups significantly reduced their levels of grief (Fig 4). Kovac and Range [55] compared in an RCT a two-week profound writing condition with a two-week trivial writing condition (*N* = 42). Time since bereavement was on average 13.26 years in the intervention and 11.95 years in the comparison group. Participants in the intervention group were asked to write for 15 minutes about events and emotions surrounding their loss, whereas participants in the comparison group were asked to describe neutral events such as their bedroom. There were no significant differences between the two groups with regard to the uncomplicated grief measure used.

However, with regard to suicide-specific aspects of grief, Kovac and Range [55] found significant reductions in the intervention group in the time between the post- and 6-month follow-up assessments as opposed to an active comparison group. This finding holds for the total score of the measure, but not for any of the subscales.

## Discussion

This systematic review provides an overview of the effects of intervention programs on grief of people bereaved by the suicide of a loved one. Studies which focused on uncomplicated grief, suicide-specific aspects of grief, and complicated grief were included. The evidence available from this systematic review provides important insight into current research gaps and has practical implications. Overall, although a remarkable proportion of the population is affected by the suicide of a loved one and is therefore at elevated risk of experiencing complicated courses of grief, only seven intervention studies were identified that were eligible for our systematic review. All of them were secondary interventions and five studies (71.4%) showed a reduction in grief intensity for at least one measure.

Of the five studies focusing on uncomplicated grief, results were mixed as three studies showed positive effects [52,54,56] and two did not [53,55]. Due to the weak methodological quality of those studies showing some positive evidence, the results should be interpreted with caution and seen as preliminary. One study [55] investigated the effect of a secondary intervention on suicide-specific aspects of grief. It has been found that participants who wrote about their bereavement experiences four times for 15 minutes over a period of two weeks reported a greater decrease of suicide-specific aspects of grief than people randomized to an attentional control condition. It can be concluded that a rather short and easy to implement intervention based on the writing paradigm developed by Pennebaker, 1986 [64] has an effect on aspects of grief specific to losing a significant other to suicide. Difficult emotions in relation to the traumatic death might be inhibited or suppressed in people bereaved by suicide or concealed as a consequence of stigmatization and lower level of support from others. Being invited to write openly in a safe environment about emotions and thoughts surrounding the suicide might have resulted in a reduction of suicide-specific aspects of grief. These results are in line with two other studies that found writing interventions to be effective in reducing the grief of bereaved individuals [65,66].

The effectiveness of secondary interventions on complicated grief in people bereaved by suicide was only investigated in two studies. One of the two randomized-controlled studies [57] with the highest level of methodological quality in this systematic review did not find any intervention effect on complicated grief. This result is in line with a meta-analysis which also found no significant overall effect of preventive interventions on complicated grief [67]. However, the second study [51] found that a cognitive-behavioral intervention was effective in the prevention of complicated grief in a subset of participants with high levels of suicidal ideation at the beginning of the study. The result that the same intervention was not effective in a sample of people bereaved by suicide in general [30] but only in a subset with high levels of suicidal ideation [51] leads to the conclusion that the effectiveness of the intervention depends on the risk level of the participants. 22% of the sample suffered from suicidal ideation three months after the loss. These suicide ideators showed significantly higher levels of neuroticism as well as lower levels of mastery and self-esteem compared to non-ideators at the beginning of the study. Furthermore they had been significantly more often diagnosed as depressed (46.2 vs. 20.2%) or anxious (38.5 vs 16.0%) in the past and had attempted suicide more often than non-ideators (18.5 vs 2.1%). They had also more often lost a child or spouse to suicide than non-ideators and showed less favorable bereavement outcomes three months post-loss with significantly higher levels of complicated grief and depression. An intervention that is based on the cognitive-behavioral concept of complicated grief [68] and contains elements of psychoeducation, enhancement of emotional processing, family communication, problem solving skills, and consolidating resources of support seems to be a promising method for preventing complicated grief in a high risk group of people bereaved by suicide. However, these results should be seen as preliminary as they were derived from post-hoc subgroup analyses. They need to be replicated in a study that randomizes people with high levels of suicidal ideation to a treatment and comparison group. Furthermore, it seems to be a promising strategy with regard to the prevention of complicated grief to provide secondary intervention to high-risk participants only. The risk screening might be based on higher levels of grief intensity but also on known risk factors for complicated grief such as: insecure attachment, preexisting mood and anxiety disorders, the nature of the relationship to the deceased, and the resources and support available following the death [36].

Considering all results, there seems to be a tendency for people bereaved by suicide to benefit from secondary intervention programs which is in line with evidence from meta-analyses and reviews for the bereaved in general that show either small positive effects for secondary interventions in the short term [8,69] or mixed results [47]. Surprisingly there is only a small number of intervention studies and none of them evaluated any tertiary interventions designed for individuals already suffering from complicated grief. Especially people bereaved by suicide are vulnerable to developing complicated grief [27–29,55] and the very is not only related to several negative mental and physical health outcomes [24,32–37], but is also a strong predictor of suicidal ideation and behavior [28,38,40].

Therefore, people bereaved by suicide are in need of and might be especially receptive to interventions aimed at reducing their grief. As Wilson and Marshall [44] showed, there is a significant gap between the need for support in people bereaved by suicide and the provision and quality of professional support services. Whereas 94% of the participants in their study indicated a need for help in managing their grief, less than half of them received help, and of those, only 40% felt satisfied with it. Our systematic review supports this result by showing that secondary interventions are rare and tertiary interventions are missing. Effective interventions for complicated grief [66,70–73] need to be adapted to and evaluated in suicide survivor populations.

### Limitations of the included studies

There are some limitations of the included studies that must be taken into account when interpreting the results of our systematic review. First, overall methodological quality of the included studies was low as the two studies with the highest methodological quality [51,57] fulfilled only four of six quality criteria. The included studies were especially weak with regard to selection bias and blinding. Participants were not very likely to be representative of the population of suicide survivors because they had referred themselves for study participation. Additionally, two studies used a non-randomized study design [54,56]. Therefore, the generalizability of the results is limited. Furthermore, internal validity may be threatened due to the fact that, with the exception of the Kovac & Range [55] study, outcome assessors were not blinded. Only one study [57] used intention-to-treat analyses whereas all other studies based their analyses on completer analyses. This may have led to a biased estimate of treatment effects [74]. Overall the results should be considered exploratory since only two studies [55,57] have adjusted analysis for multiple testing. Moreover, because of the small sample sizes, it cannot be ruled out that small treatment effects were overseen due to the low power of the tests. Second, complicated grief was measured on a symptom level only. None of the two studies [51,57] using the Inventory of Traumatic Grief used cut-off values to separate participants with clinically relevant grief intensity from others even though such threshold values are available. Also, the functional impairment and time criteria that need to be fulfilled to diagnose complicated grief as a mental disorder [23,25] were not taken into account. Third, intervention duration was rather short in all of the studies, ranging from two weeks to four months, and all but one intervention [55] was implemented in a group setting. This may have led to smaller treatment effects as there is some evidence that a longer intervention duration and individual grief therapy might be more effective [75]. Furthermore, nearly all interventions were implemented within the first year after loss. Therefore, intervention effects may interfere with the "natural" grieving processes [47] making strong treatment effects less likely. Fourth, the results of this systematic review are limited to a white, female, middle-aged population with tight familiar relationships to the deceased. Interventions targeting children and adolescents as well as the elderly were missing completely. Younger and older people may experience more complications of their bereavement process [26,37] and may therefore be more difficult to treat [75]. Last, since long-term follow-up assessments were largely missing it remains unclear whether intervention effects remain stable over time.

### Limitations of the systematic review

Searching for only English language articles may have led to the exclusion of relevant studies published in other languages. Also a broader search string might have led to more articles. Furthermore, including only studies published in peer-reviewed journals might have led to missing important knowledge from unpublished “gray” literature, which might result in publication bias. However, by including peer-reviewed articles a minimum of methodological quality was ensured. This systematic review was also not limited to RCT, which limits the level of evidence. Due to the small number of RCTs we decided to include all studies investigating any kind of intervention to gain as much insight as possible, thus providing directions for future research.

### Implications for research and clinical practice

First, future research should focus on tertiary interventions, i.e. including participants diagnosed with complicated grief in clinical interviews, or at least screened for elevated symptoms of complicated grief with questionnaires providing cut-off values. Grief interventions which have already been shown to be effective in bereaved persons in general should be adapted to and investigated in this specific population. Second, because one promising study in our review showed that secondary interventions might be effective in preventing complicated grief if they are addressed to a subset of people at higher risk of complications of their bereavement process, future studies focusing on the prevention of complicated grief should include high risk participants only. Third, methodologically sound randomized controlled trials that adjust for multiple testing, conduct sample size calculations, intention-to-treat analyses, and long-term follow-up assessments are needed. Additionally, outcome assessors should be blinded and more effort should be made to include a representative sample of the population of suicide survivors.

People bereaved by suicide constitute a remarkable proportion of the population with an increased risk of experiencing complicated courses of grief. Untreated complicated grief might pose an independent risk factor for suicidal thoughts and action and could in turn contribute to the family transmission of suicidal behavior. General practitioners and mental health professionals, in particular, should screen patients who have lost a loved one to suicide for complicated grief and suicidal ideation. High-risk patients should then be referred to psychotherapists.

### Conclusions

The aim of this systematic review was to evaluate the effects of interventions on grief for people bereaved by the suicide of a loved one. Studies investigating grief interventions for suicide survivors are rare and the results of these studies need to be interpreted with caution due to notable methodological limitations. Nevertheless, the preliminary results indicate some positive effects of interventions in reducing grief intensity and suicide-specific aspects of grief. Study results regarding complicated grief are less promising. Only one out of two studies found that a cognitive-behavioral intervention was effective in the prevention of complicated grief but only for a subset of participants with high levels of suicidal ideation at the beginning. This suggests that the effectiveness of a grief intervention might depend on the risk level of the participants. Further research is necessary in order to adapt and evaluate effective grief interventions for people bereaved by suicide that are in particular need of support.

## Supporting information

S1 PRISMA Checklist PRISMA checklist. (DOC)

S1 File List of excluded full-text articles. (DOCX)
