# Supplementary material for: Grief interventions for people bereaved by suicide: A systematic review
Source: PLoS One. 2017 Jun 23;12(6):e0179496. doi: 10.1371/journal.pone.0179496 (PMC5482439; doi:10.1371/journal.pone.0179496)
Supplement: S1 File — (DOCX) [file pone.0179496.s002.docx]

**Grief interventions for people bereaved by suicide: A systematic review.**

**Reasons for exclusion of full-text articles**

1. Bacqué M-F, Haegel C, Silvestre N. Résilience de l’enfant endeuillé. = Resilience of bereaved children. Prat Psychol. 2000;1:23–33.

Reason for exclusion: Not written in English

1. Barlow CA, Morrison H. Survivors of suicide. Emerging counseling strategies. J Psychosoc Nurs Ment Health Serv. 2002;40(1):28–39.

Reason for exclusion: Descriptive Study

1. Battle AO. Group therapy for survivors of suicide. Crisis J Crisis Interv Suicide Prev. 1984;5(1): 45–58.

Reason for exclusion: Descriptive Study

1. Braiden HJ, McCann M, Barry H, Lindsay C. Piloting a therapeutic residential for children, young people and families bereaved through suicide in Northern Ireland. Child Care Pract. 2009 Apr;15(2):81–93.
   Reason for exclusion: Descriptive Study
2. Campbell FR, Cataldie L, McIntosh J, Millet K. An Active Postvention Program. Crisis J Crisis Interv Suicide Prev. 2004;25(1):30–2.
   Reason for exclusion: Descriptive Study
3. Campbell FR. The influence of an active postvention on the length of time elapsed before survivors of suicide seek treatment. Diss Abs Int A Hum Soc Sci. 2002;63:753.

Reason for exclusion: Not published in peer-reviewed journals

1. Catone WV, Schatz MT. The crisis moment: A school’s response to the event of suicide. Sch Psychol Int. 1991 Apr;12(1–2):17–23.
   Reason for exclusion: Descriptive Study
2. Cerel J, Campbell, FR. Suicide survivors seeking mental health services: A preliminary examination of the sole of an active postvention model. Suicide Life-Threat Behav*.* 2008;38(1): 30–4.

Reason for exclusion: No grief measure used

1. Clark, SE, Goldney RD. Grief reactions and recovery in a support group for people bereaved by suicide. Crisis J Crisis Interv Suicide Prev. 1995;16(1):27–33.

Reason for exclusion: Qualitative Study

1. Cohen JA, Mannarino AP, Knudsen K. Treating childhood traumatic grief: A pilot study. J Am Acad Child Adolesc Psychiatry. 2004 Oct;43(10):1225–33.
   Reason for exclusion: Contains a mixed group of bereaved participants
2. Comans T, Visser V, Scuffham P. Cost effectiveness of a community-based crisis intervention program for people bereaved by suicide. Crisis. 2013;34(6):390–7.

Reason for exclusion: No grief measure used

1. Daigle MS, Labelle RJ. Pilot evaluation of a group therapy program for children bereaved by suicide. Crisis J Crisis Interv Suicide Prev. 2012;33(6):350–7.
   Reason for exclusion: Descriptive Study
2. Dane B. Counseling Bereaved Middle-Aged Children - Parental Suicide Survivors. Clin Soc Work J. 1991 SPR;19(1):35–48.
   Reason for exclusion: Descriptive Study
3. de Castro S, Guterman JT. Solution-focused therapy for families coping with suicide. J Marital Fam Ther. 2008 Jan;34(1):93–106.
   Reason for exclusion: Descriptive Study
4. De fauw N, Andriessen K. Networking to support suicide survivors. Crisis J Crisis Interv Suicide Prev. 2003;24(1):29–31.
   Reason for exclusion: Descriptive Study
5. de Groot M, de Keijser J, Neeleman J, Kerkhof A, Nolen W, Burger H. Cognitive behaviour therapy to prevent complicated grief among relatives and spouses bereaved by suicide: Cluster randomised controlled trial. BMJ. 2007;334(7601):994.
   Reason for exclusion: Same intervention as De Groot et al. 2010
6. Feigelman B, Feigelman W. Surviving after suicide loss: The healing potential of suicide survivor support groups. Illn Crisis Loss. 2008;16(4):285–304.
   Reason for exclusion: Descriptive Study
7. Feigelman B, Feigelman W. Suicide survivor support groups: Comings and goings, Part I. Illn Crisis Loss. 2011;19(1):57–71.

Reason for exclusion: Cross-sectional Study

1. Feigelman B, Feigelman W. Suicide survivor support groups: Comings and goings, part II. Illn Crisis Loss. 2011;19(2):165–85.
   Reason for exclusion: Descriptive Study
2. Feigelman W, Gorman BS, Beal KC, Jordan JR. Internet support groups for suicide survivors: A new mode for gaining bereavement assistance. Omega J Death Dying. 2008;57(3):217–43.
   Reason for exclusion: Cross-sectional Study
3. Gaffney DA, Jones ET, Dunne-Maxim K. Support groups for sibling suicide survivors. Crisis J Crisis Interv Suicide Prev. 1992;13(2):76–81.
   Reason for exclusion: Descriptive Study
4. Groos AD, Shakespeare-Finch J. Positive Experiences for Participants in Suicide Bereavement Groups: A Grounded Theory Model. Death Stud. 2013;37(1):1–24.
   Reason for exclusion: Qualitative Study
5. Hajal F. Post-Suicide Grief Work in Family-Therapy. J Marriage Fam Couns. 1977;3(2):35–42.
   Reason for Exclusion: Descriptive Study
6. Hatton, CL, Valente, SM. Bereavement group for parents who suffered a suicidal loss of a child. Suicide Life Threat Behav. 1981;11(3):141–50.

Reason for Exclusion: Descriptive Study

1. Hawton K, Sutton L, Simkin S, Walker D-M, Stacey G, Waters K, et al. Evaluation of a Resource for People Bereaved by Suicide. Crisis- J Crisis Interv Suicide Prev. 2012;33(5):254–64.
   Reason for Exclusion: Qualitative Study
2. Hazell P, Lewin T. An evaluation of postvention following adolescent suicide. Suicide Life Threat Behav. 1993:23:101-9

Reason for Exclusion: No grief measure used

1. Hazell P. Postvention after teenage suicide: an Australian experience. J Adolescence. 1991;14:335-42.

Reason for Exclusion: Descriptive Study

1. Hollander EM. Cyber community in the valley of the shadow of death. J Loss Trauma. 2001 Jun;6(2):135–46.
   Reason for Exclusion: Descriptive Study
2. Juhnke GA, Shoffner MF. The Family Debriefing Model: An adapted Critical Incident Stress Debriefing for parents and older sibling suicide survivors. Fam J. 1999 Oktober;7(4):342–8.
   Reason for Exclusion: Descriptive Study
3. Kaslow NJ, Ivey AZ, Berry-Mitchell F, Franklin K, Bethea K. Postvention for African American families following a loved one’s suicide. Prof Psychol Res Pract. 2009 Apr;40(2):165–71.
   Reason for Exclusion: Descriptive Study
4. Mauk G, Rodgers P. Building Bridges Over Troubled Waters - School-Based Postvention with Adolescent Survivors of Peer Suicide. Crisis Interv Time-Ltd Treat. 1994;1(2):103–23.
   Reason for Exclusion: Descriptive Study
5. Mitchell AM, Evanczuk K, Lucke J. Evaluation of critical incident stress debriefing for survivors of suicide: Preliminary results. Proceedings of the University of Hawaii’s Clinical Research and the Managed Care Environment Conference; Oahu, HI: University of Hawaii; 1999. Mar

Reason for Exclusion: Not published in peer-reviewed journals

1. Mitchell AM, Kim Y. Debriefing approaches with suicide survivors. Proceedings of the Suicide Survivor Research Workshop, sponsored by the National Institute of Mental Health (NIMH) and the American Foundation for Suicide Prevention (AFSP); Bethesda, MD. 2003. May

Reason for Exclusion: Not published in peer-reviewed journals

1. Mitchell AM, Wesner S, Garand L, Gale DD, Havill A, Brownson L. A Support Group Intervention for Children Bereaved by Parental Suicide. J Child Adolesc Psychiatr Nurs. 2007 Feb;20(1):3–13.
   Reason for Exclusion: Descriptive Study
2. Murphy SA. Parent bereavement stress and preventive intervention following the violent deaths of adolescent or young adult children. Death Stud. 1996 Oct;20(5):441–52.
   Reason for Exclusion: Descriptive Study
3. Murphy SA. A bereavement intervention for parents following the sudden, violent deaths of their 12-28-year-old children: description and applications to clinical practice. Can j nurs res. 1997;29(4):51–72.

Reason for Exclusion: Contain a mixed group of bereaved participants

1. Murphy SA, Johnson C, Cain KC, Das Gupta A, Dimond M, Lohan J. Broad-spectrum group treatment for parents bereaved by the violent deaths of their 12- to 28-year-old children: A randomized controlled trial. Death Stud. 1998 May;22(3):209–35.
   Reason for Exclusion: Contain a mixed group of bereaved participants
2. Murphy SA, Lohan J, Dimond M, Fan JJ. Network and mutual support for parents bereaved following the violent deaths of their 12- to 28-year-old children: A longitudinal, prospective analysis. J Pers Interpers Loss. 1998 Dec;3(4):303–33.
   Reason for Exclusion: Contain a mixed group of bereaved participants
3. Pfeffer CR, Jiang H, Kakuma T, Hwang J, Metsch M. Group intervention for children bereaved by the suicide of a relative. J Am Acad Child Adolesc Psychiatry. 2002 May;41(5):505–13.
   Reason for Exclusion: No grief measure used
4. Pietila M. Support groups: a psychological or social device for suicide bereavement? Br J Guid Couns. 2002 Nov;30(4):401–14.
   Reason for Exclusion: Descriptive Study
5. Poijula S, Dyregrov A, Wahlberg KE, Jokelainen J. Reactions to adolescent suicide and crisis intervention in three secondary schools. Int J Emerg Ment Health. 2001;3(2):97–106.
   Reason for Exclusion: Cross-sectional Study
6. Poijula S, Wahlberg KE, Dyregrov A. Adolescent suicide and suicide contagion in three secondary schools. Int J Emerg Ment Health. 2001;3(3):163–8.

Reason for Exclusion: Descriptive Study

1. Renaud C. Bereavement after a suicide: a model for support groups. In: Mishara BL, editor. The impact of suicide. New York: Springer Publishing Co.; 1995: 52-63.

Reason for Exclusion: No grief measure used

1. Rogers J. Help for families of suicide: Survivors Support Program. Can J Psychiatry. 1982;27(6):444–9.

Reason for Exclusion: Descriptive Study

1. Sandler  [I](https://www.ncbi.nlm.nih.gov/pubmed/?term=Sandler%20I%5BAuthor%5D&cauthor=true&cauthor_uid=27094109), Tein JY, [Wolchik S](https://www.ncbi.nlm.nih.gov/pubmed/?term=Wolchik%20S%5BAuthor%5D&cauthor=true&cauthor_uid=27094109), [Ayers TS](https://www.ncbi.nlm.nih.gov/pubmed/?term=Ayers%20TS%5BAuthor%5D&cauthor=true&cauthor_uid=27094109). The effects of the family bereavement program to reduce suicide ideation and/or attempts of parentally bereaved children six and fifteen years later. Suicide Life Threat Behav. 2016 Apr;46 Suppl 1:32-8.

Reason for Exclusion: Contain a mixed group of bereaved participants

1. Sandor MK., Walker LO, Sands D. Competence-building in adolescents, Part II: Community intervention for survivors of peer suicide. Issues Compr Pediatr Nurs. 1994;17(4):197–209.

Reason for Exclusion: No grief measure used

1. Sanford R, Cerel J, McGann V, Maple M. Suicide loss survivors' experiences with therapy: implications for clinical practice. Community Ment Health J. 2016 Jul;52(5):551-8.

Reason for Exclusion: Cross-sectional Study

1. Scocco P, Frasson A, Costacurta A, Pavan L. SOPRoxi - A research-intervention project for suicide survivors. Crisis- J Crisis Interv Suicide Prev. 2006;27(1):39–41.
   Reason for Exclusion: Descriptive Study
2. Seguin M, Lesage A, Kiely MC. Parental bereavement after suicide and accident: A comparative study. Suicide Life Threat Behav. 1995 WIN;25(4):489–98.
   Reason for Exclusion: Not written in English
3. Séguin M. Le deuil après un suicide: facteurs psycho-sociaux et programme d’intervention. = Bereavement after suicide: Psycho-social factors and intervention program. Psychol Medicale. 1990 Apr;22(5):377–9.
   Reason for Exclusion: Not written in English
4. Spino E, Kameg KM, Cline TW, Terhorst L, Mitchell AM. Impact of social support on symptoms of depression and loneliness in survivors bereaved by suicide. Arch Psychiatr Nurs. 2016 Oct;30(5):602-6.

Reason for Exclusion: Cross-sectional Study

1. Stepakoff S. From destruction to creation, from silence to speech: Poetry therapy principles and practices for working with suicide grief. Arts Psychother. 2009 Apr;36(2):105–13.
   Reason for Exclusion: Cross-sectional Study
2. Supiano KP. Sense-Making in Suicide Survivorship: A Qualitative Study of the Effect of Grief Support Group Participation. J Loss Trauma. 2012;17(6):489–507.
   Reason for Exclusion: Qualitative Study
3. Trimble T, Hannigan B, Gaffney M. Suicide postvention; Coping, support and transformation. Ir J Psychol. 2012 Jun;33(2–3):115–21.
   Reason for Exclusion: Qualitative Study
4. Visser VS, Comans TA, Scuffham PA. Evaluation of the Effectiveness of a Community-Based Crisis Intervention Program for People Bereaved by Suicide. J Community Psychol. 2014 Jan;42(1):19–28.
   Reason for Exclusion: No grieve measure used
5. Walijarvi CM, Weiss AH, Weinman ML. A traumatic death support group program: applying an integrated conceptual framework. Death Stud. 2012 Feb;36(2):152–81.
   Reason for Exclusion: Descriptive Study
6. Watson SL. The grief process of suicide survivors: a study on the effectiveness of psychotherapy and support group interventions. Diss Abs Int B Sci Eng. 1992;53:1620.

Reason for Exclusion: Not published in peer-reviewed journals

1. Wilkens NT. Christian-based counseling for the suicide survivor: a guide for pastoral therapy. JPCC. 2003;57(4):387–94.

Reason for Exclusion: Descriptive Study

1. Wrobleski A. The suicide survivors grief group. Omega- J Death Dying. 1985;15(2):173–84.

Reason for Exclusion: Descriptive Study
